# Supplementary figures and images for: Nonpolar residues in the presumptive pore‐lining helix of mechanosensitive channel MSL10 influence channel behavior and establish a nonconducting function
Source: Plant Direct. 2018 Jun 5;2(6):e00059. doi: 10.1002/pld3.59 (PMC6261518; doi:10.1002/pld3.59)

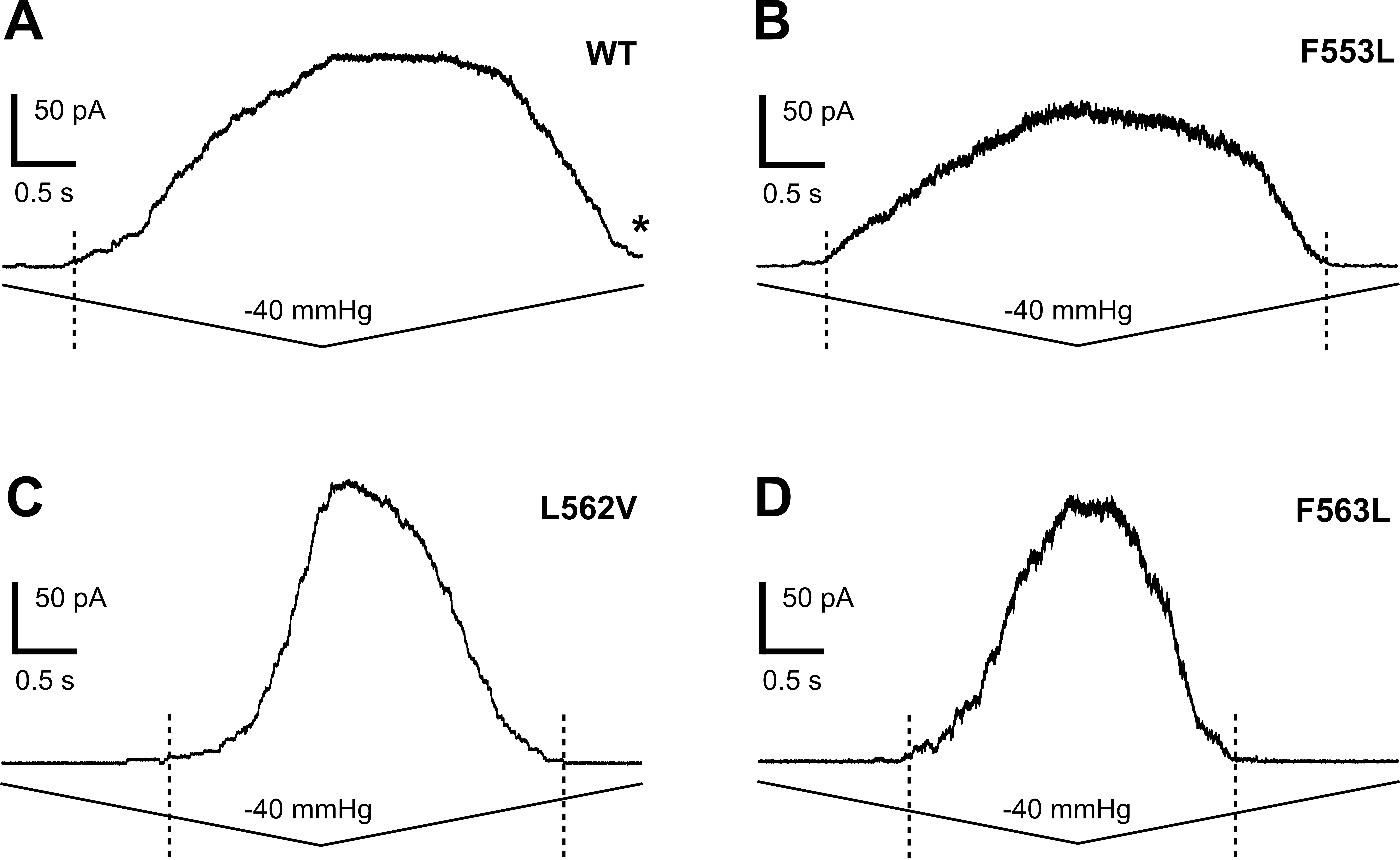

Supplement: Supplementary file 1 [file PLD3-2-e00059-s001.png]
