# Supplementary material for: Nonpolar residues in the presumptive pore‐lining helix of mechanosensitive channel MSL10 influence channel behavior and establish a nonconducting function
Source: Plant Direct. 2018 Jun 5;2(6):e00059. doi: 10.1002/pld3.59 (PMC6261518; doi:10.1002/pld3.59)
Supplement: Supplementary file 2 [file PLD3-2-e00059-s002.pdf]

Reviewer #1 :

The authors demonstrated that mutations in non-polar residues, especially F553 L554 and G556 in the pore-lining helix of MSL10 affect channel conductance, activation threshold, and gating hysteresis, and that channel function is not required for apoptosis signalling in Arabidopsis. They analysed mutants of MSL10 with Xenopus oocyte patch clamp technique. The data shown in this manuscript support well the conclusion, however, it needs to be amended. Here are suggestions to improve the manuscript.

Major points:

1. Result, page 10, lines 215-217. Regarding G556 residue as a possible kink of MSL10, it is too preliminary to conclude that TM6 is bent at this residue like TM3 of EcMscS. Predicted structure with a computational modelling is not significantly bent, and the results of the mutation are different between MscS and MSL10, for example, G113 of MscS is important for the inactivation, but not MSL10 in the oocyte membrane although MSL10 shows inactivation in the plant native membrane, thus the role of glycine could be different. The authors should be more careful to describe the conclusion.

We agree with the reviewer's statements above, but do not believe that we make any of these conclusions. We state that "Our results establish that G556 plays a key role in the function of MSL10 and are consistent with the prediction that there is bending at this residue within the helix and that mobility at this site is important for the conformational changes associated with channel opening." We do not conclude that it plays a role analogous to that of G113 in MscS, nor that it plays any role in inactivation, neither here nor in the Discussion.

2. Result, page 12, Figure 5. The gating hysteresis of MSL10 is an interesting feature for readers. The authors should show raw patch-clamp traces for the WT, F553L, L562V, and F563L in the Figure 5. The raw traces may go to supplemental information because of the limited space.

We have added the suggested supplementary figure.

3. Result, page 12, lines 257-258. The channel-channel interaction of MSL10 is not trivial. The authors should provide a possible explanation about the difference between F553L and F563L about the result in the Figure 5A and B.

We deleted the statement "Thus, some aspect of channel-channel interaction may be altered in MSL10 F563L", which is speculation and doesn't really belong in the results section anyway.

4. Discussion, page 16, lines 329-334, page 17, lines 351-355. I agree that no-mechanosensitive activities of F553V and G556V are keys to understand the gating mechanism of MSL10, and that apoptosis signalling is separated from channel function as shown in Figure 6. However, to conclude that they are loss-of-function mutations of MSL10 (surprisingly, non-conductive channels), I am wondering if the pore size of MSL10 is really determined by the side chain at the F553 residue. If it is packed more closely with small chain, activation threshold should be increased in this mutant because it is going to be more difficult to change the conformation. However, the activation threshold of F553L seems to be similar to WT MSL10 as shown in Figure 5A. The author should describe more carefully this result in the discussion.

We have edited the discussion to say:

"In this model, the flickery behavior of MSL10 F553L is explained by a steric mismatch between the mutated residue and its interaction partner from the adjacent pore-lining domain. The F553V mutant would have no channel activity as the non-polar side chain could not create even a partially open pore. There may be a similar interaction that stabilizes the closed state, though F553L has a tension threshold similar to that of the WT MSL10 channel, suggesting that this explanation might be too simple. If reducing the size of the side chain at F553 stabilizes the closed pore, one would expect the flickery F553L channel to be harder to activate."

Minor points:
1. The word "lesions" is not easy to understand the meaning immediately for readers. It is better to use "mutations" in this manuscript.

We made this replacement throughout the manuscript.

2. Result, page 12, Figure 5. It is better to add WT MSL10 data for the comparison in the Figure 5C.

There is no result for MSL10, as indicated by the empty dataset in the chart in Figure 5C. We have added the following text to the results section to make this more clear: "It is not possible to calculate an open/closed ratio for WT MSL10. In almost every case, one or more channels are still open when all

applied tension is released. As a result, the closing threshold tension cannot be calculated.”

Reviewer #2 :

In the manuscript "Nonpolar residues in the presumptive pore-lining helix of mechanosensitive channel MSL10 influence channel behavior and confirm a non-conducting function" the authors goal is to determine the role of 8 residues in the pore lining helix of MSL10 from Arabidopsis thaniana. To investigate the role of these residues the authors utilized point mutations, patch clamp electrophysiology on oocytes, and used in vitro assays to investigate non-mechanosensitive behavior. The authors definitively determined in addition to the mechanosensation that MSL10 gates in response to, it has a significant role in programed cell death that is separate from the channel behavior.

In general, I found this paper to be a riveting read where the authors answered each question I had about MSL10 as soon as I had formed it in my mind. I found the experimental design to be complete to have utilized the correct controls for experimental variation. In particular I found the use of the pipettes with similar resistance to control for patch variety to be a clever method to answer the questions of gating threshold without an internal control.

Major Corrections
None

Moderate Corrections
1- In the section beginning on Line 167 the goal is to investigate how F553 is involved in gating in response to mechanical tension in MSL10.
a. The authors state that they are going to create lesions at F544, F553, and F563 to smaller nonpolar residues (Line 173-4), they then create a F553W lesion which is neither small nor nonpolar. I believe they were using F553W as a conservative mutation, would be possible to clarify your thoughts behind this point mutation.

We added “and to introduce a series of increasingly disruptive changes at F553” to this sentence to better describe our intentions with these point mutations.

b. In Figure 2C one of the traces is labeled as F554V, based on the text I

believe this to be a typo for F544V. Please correct the text or the Figure so that they are in reference to the same mutation. The confusion on this figure was exacerbated by the lack of GFP image for the corresponding mutation.

We have fixed the typo in Figure 2C.

2- Figure 4B: In the figure caption the gray box indicates the region where I554S is statistically different from WT MSL10, however the current/voltage plot for WT is missing in this plot. I found it difficult to predict where the WT curve would lie in this figure, would it be possible to add this to the graph? However, in the event that this would mask the data the addition of a statement to the figure caption mentioning where this curve is expected would be acceptable.

We added to the figure caption "L548V, A550L, I554V, and L562V do not differ from the WT."

3- In the section beginning on line 239, studying the hysteresis of MSL10 mutations.

a. In Panel A the addition of colored lines that correlate with the colored squares and triangles would assist the reader in understanding which of the lines correlates to the WT and which correlates to MSL10 F553L

We made this change.

b. In Panel C: It was unclear how the Open/Close Threshold Ratio was obtained. In the figure the Ratio for WT was particularly confusing, based on the introduction my understanding is that MSL10 exhibits strong hysteresis which would lead to the channels closing at a lower tension than the second channels opening. In the data the WT ratio is zero (or blank), while the data is not collectable a further explanation of how the data is collected and what is observed for the WT channel would be appreciated. My guess is that the open to close ratio is greater than 10, showing that the opening tension is higher than the closing tension but the figure implies differently.

We have added the following text to the results section to better explain the data: "It is not possible to calculate an open/closed ratio for WT MSL10. In almost every case, one or more channels are still open when all applied tension is released. As a result, the closing threshold tension cannot be calculated. However, the fact that all three MSL10 variants did not show this

behavior made it possible to calculate an open/close ratio, using patches with 5-600 channels (Figure 5C).

Minor Corrections
1- On line 73 there is a typo in the reference, it reads reference 2217.

It was meant to read 22, 17—this has been fixed.

2- On line 278-9: The As shown in fig 6A... It is unclear what 'As' refers to, so potentially a typo or please clarify the statement

Deleted the word "The" so sentence starts with "As shown . . ."

3- Line 305: It would be clearer if the sentence read 'If this were the case" than "In this case" as the manuscript currently reads.

We made this change on line 313.

4- Line 360: Would it be possible to reference the figure (Figure 4) where this data can be found?

Done.

5- Line 368: Would it be possible to reference the figure (Figure 5), where this data can be found?

Done.

6- In Table 1 the addition of a column containing the fold difference from wildtype will highlight the differences in each of these lesions in comparison to WT

Done.
